# Suppressing endoplasmic reticulum stress-related autophagy attenuates retinal light injury

**DOI:** 10.18632/aging.103846

**Published:** 2020-08-28

**Authors:** Jing-Yao Song, Bin Fan, Lin Che, Yi-Ran Pan, Si-Ming Zhang, Ying Wang, Victoria Bunik, Guang-Yu Li

**Affiliations:** 1 Department of Ophthalmology, Second Hospital of Jilin University, Changchun, China; 2 Department of Hemooncology, Second Hospital of Jilin University, Changchun, China; 3 A.N.Belozersky Institute of Physico-Chemical Biology, Lomonosov Moscow State University, Moscow, Russia

**Keywords:** oxidative stress, ER stress, autophagy, AMD, PERK

## Abstract

Excessive light exposure is a principal environmental factor, which can cause damage to photoreceptors and retinal pigment epithelium (RPE) cells and may accelerate the progression of age-related macular degeneration (AMD). In this study, oxidative stress, endoplasmic reticulum (ER) stress and autophagy caused by light exposure were evaluated *in vitro* and *in vivo*. Light exposure caused severe photo-oxidative stress and ER stress in photoreceptors (661W cells) and RPE cells (ARPE-19 cells). Suppressing either oxidative stress or ER stress was protective against light damage in 661W and ARPE-19 cells and N-acetyl-L-cysteine treatment markedly inhibited the activation of ER stress caused by light exposure. Moreover, suppressing autophagy with 3-methyladenine significantly attenuated light-induced cell death. Additionally, inhibiting ER stress either by knocking down PERK signals or with GSK2606414 treatment remarkably suppressed prolonged autophagy and protected the cells against light injury. *In vivo* experiments verified neuroprotection via inhibiting ER stress-related autophagy in light-damaged retinas of mice. In conclusion, the above results suggest that light-induced photo-oxidative stress may trigger subsequent activation of ER stress and prolonged autophagy in photoreceptors and RPE cells. Suppressing ER stress may abrogate over-activated autophagy and protect the retina against light injury.

## INTRODUCTION

Age-related macular degeneration (AMD) is a degenerative retinal disease, which often occurs in the elderly and causes irreversible loss of central vision. Indeed, excessive and prolonged light exposure may damage the retina and is an environmental factor that can accelerate AMD [1]. With the rapid development of technology, many electronic devices with screens, and ophthalmic equipment with intensive illumination, have become widely used. Therefore, an increasing amount of attention has been focused on issues of light pollution and retinal light damage. Among the molecular mechanisms involved in light-induced retinal damage, photochemical damage is regarded as the main culprit [2]. Excessive photons from visible light exposure are absorbed by opsin in photoreceptor cells or melanosomes in pigment epithelial cells, causing a series of photochemical reactions, which may further trigger programmed cell death [3]. However, the exact molecular mechanism of light-induced retinal injury remains unclear.

Two stereoisomers, 11-cis-retinal and all-trans-retinal, exist in the retina and play roles in the visual cycle. A photon from visible light triggers cis–trans isomerization, converting the bent chromophore into a straight conformation, causing a separation between the two stereoisomers [4, 5]. When oxygen is present and upon photoexcitation by light, all-transretinal is a potent photosensitizer that generates reactive oxygen species (ROS), such as singlet oxygen, superoxide and hydrogen peroxide [6, 7]. The excessive ROS may lead to oxidative-stress damage in both photoreceptors and RPE cells following disk phagocytosis [3, 8]. There is growing evidence that photo-oxidative reactions might be an important step in triggering the death cascade in light-damaged retinal neurons [2, 9–12].

The endoplasmic reticulum (ER) is an important organelle that mediates protein synthesis, processing and transport and participates in maintaining homeostasis of the intracellular environment [13, 14]. In the process of protein folding, resident protein disulfide isomerases (PDI), endoplasmic reticulum oxidoreduction 1 (ERO1) and glutathione (GSH) cooperate as a chaperone-like assisted mechanism to prevent and correct aberrant disulfide bonds [15]. Previous studies have shown that excessive intracellular ROS may lead to depletion of the GSH pool and compromise the function of PDI, which disrupts the folding process of proteins in the ER and produces a massive amount of misfolded proteins [16, 17]. However, the excessive accumulation of misfolded proteins in the ER may trigger an unfolded-protein response (UPR), which may enhance protein folding ability, as well as the homeostasis of protein translation and accelerate protein degradation to recover ER function [18]. Normally, the UPR is activated by 3 transmembrane ER stress sensors: activating transcription factor 6 (ATF6), inositol-requiring enzyme 1 (IRE1) and protein kinase RNA-like ER kinase (PERK). When ER stress happens, ATF6 is transported to the Golgi in the form of vesicles and is then cleaved by protease [sites 1 and 2 protease (S1P and S2P)] to produce a transcriptionally active polypeptide [19]; IRE1 is phosphorylated and causes the activation of endoribonuclease, splicing of the 26-nucleotide (nt) sequence from the X-box binding protein (XBP1) messenger ribonucleic acid (mRNA) and production of functional XBP1(S), which is transferred to the nucleus and activates transcription of the genes encoding the ER chaperone [20]; PERK is activated by phosphorylation, which in turn phosphorylates eukaryotic translation initiation factor 2 (EIF2) to inhibit protein translation and reduce protein synthesis [21]. However, phosphorylated EIF2 may selectively increase activating transcription factor 4 (ATF4) translation [22], which causes the activation of transcription factor C/EBP homologous protein (CHOP). Under physiological conditions, these three transmembrane proteins bind to the glucose regulatory protein 78kDa (Grp78; also known as BiP) in the ER lumen. Once misfolded proteins accumulate in the ER, they titrate BiP away from these sensors to cause activation of the downstream signals, which may further accelerate protein degradation, termed ER-related degradation (ERAD) [23]. ERAD is mainly composed of the following mechanisms: ubiquitin proteasome-dependent ERAD and autophagy lysosome-dependent ERAD [24].

Autophagy is an important and complex metabolic pathway in eukaryotic cells. It normally consists of four main phases: nucleation, expansion, maturation, and degradation/recycling [25]. Autophagy is often classified as basic autophagy, which exists at a relatively low level in cells, and induced autophagy, which is caused by various stresses, such as starvation, aging and inflammation [26–28]. Autophagy is a major intracellular degradation system and is responsible for the degradation of long-lived proteins, organelles and other cellular contents [29]. Moreover, autophagy is an important mechanism of ERAD and participates in the degradation of misfolded proteins and protein aggregates following ER stress [30]. However, prolonged autophagy may lead to cell death and is specifically termed autophagy-dependent cell death [31]. The role of autophagy in retinal light injury is controversial. Autophagy might be a double-edged sword among the molecular mechanisms that lead to retinal light damage. Midorikawa et al. reported that moderate autophagy combined with endosomal degradation pathway activity is neuroprotective and attenuates light-dependent retinal degeneration [32]. However, Zhang et al. showed that over-activated autophagy is detrimental to light damaged photoreceptors and that suppressing autophagy with 3-methyladenine (3MA) may protect photoreceptors against light injury [33]. Therefore, further clarifying the role of autophagy in retinal light injury is still necessary and whether autophagy activation is related to ER stress-related pathways needs further investigation.

Previous studies employing histopathological analysis of retinal sections have shown that visible light-induced damage predominantly occurs in the outer layer of the retina, especially in the layer of photoreceptor cells and pigment epithelial cells [3, 34]. Therefore, in this study the light-induced death mechanism was investigated with *in vitro* experiments using two kinds of cell lines: photoreceptor cells (661W) and pigment epithelial cells (ARPE-19). The *in vivo* experiments, specifically focused on light-induced alternations in the outer layer of the retina and the RPE layer, in order to determine the changes in the retina and RPE/choroid mixture. It was found that visible light exposure caused severe photo-oxidative-stress damage in photoreceptors and RPEs following ER stress-related autophagy and that inhibiting oxidative stress with the antioxidant N-acetyl-L-cysteine (NAC) suppressed ER stress caused by light exposure and protected cells against light damage. In addition, either directly inhibiting prolonged autophagy with 3MA or suppressing over-activated autophagy by inhibiting ER stress (knockdown PERK or treated with SAL, an ER stress inhibitor) also protected photoreceptors and RPE cells from light injury. Finally, the potent role of ER stress-related autophagy was further verified with *in vivo* experiments. This study suggests that visible light exposure may cause prolonged autophagy in photoreceptors and RPE cells and that suppressing ER stress-related autophagy may effectively protect the retina against light injury. Furthermore, this research deciphered the molecular mechanisms involved in retinal light injury, which may lay the experimental foundation for further development of neuroprotective drugs for light damage-related retinal degenerative diseases.

## RESULTS

### Light exposure induces oxidative stress in photoreceptors and RPE cells

**Figure 1:**
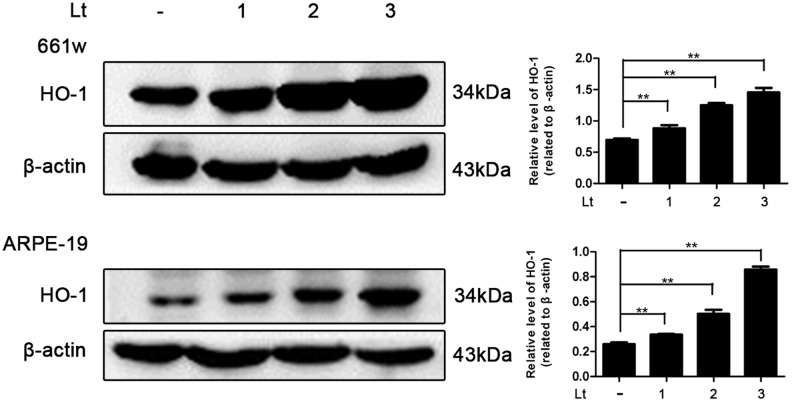
**Light exposure increases the level of HO-1 in photoreceptors and RPEs.** 661W cells/ARPE-19 cells were cultured in dark conditions or exposed to 1500 Lux light for 1–3 days. The level of HO-1 protein in the whole cell lysate was determined with western blotting, and β-actin was referenced as an internal control. Three independent experiments are conducted two weeks apart. The results are presented as the mean± SEM. n (per group) =3, **P < 0.01.

**Figure 2:**
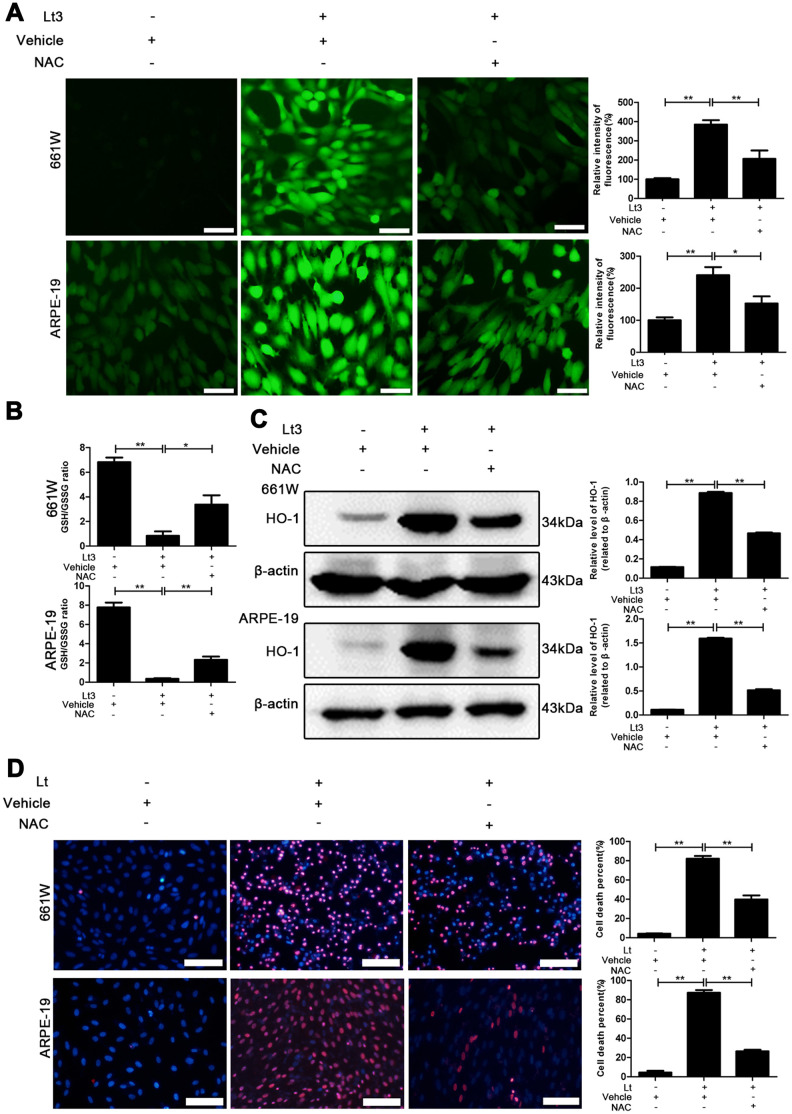
**NAC treatment suppresses light-induced oxidative stress.** 661W cells/ARPE-19 cells were pretreated with NAC (5 mM for 661W cells and 2.5 mM for ARPE-19 cells) or vehicle and cultured under light/dark conditions for 3 days. (**A**) The intracellular ROS were stained with DCFH-DA fluorescent probe identified by green fluorescence. Scale bar=50 μm. Relative fluorescence intensities were calculated and compared. (**B**) The GSH/GSSG ratio was measured with a GSH/GSSG Assay Kit. (**C**) The HO-1 level was determined with western blotting, and β-actin was referred as an internal control. (**D**) 661W cells pretreated with 5 mM NAC/vehicle were cultured under light/dark conditions for 3 days. ARPE-19 cells pretreated with 2.5 mM NAC/vehicle were cultured under light/dark conditions for 6 days. The cell death percentage was evaluated with PI/Hoechst staining. Scale bar=100μm. The percentage of cell death was calculated as PI-positive cells/total cells%. Three independent experiments are conducted two weeks apart. The results are presented as the mean± SEM. n (per group) =3, *P < 0.05, **P < 0.01.

Photo-oxidative-stress damage may be the initial step triggering neuronal death in the outer layer of the retina, the imbalance of the cellular redox status induced by light exposure was first evaluated by exposing 661W cells and ARPE-19 cells to 1500 Lux light for 1–3 days. The induced isomer of heme oxygenase, HO-1 is a protein marker that indicates cellular redox status [35] and was quantitatively determined via western blot. As shown in Figure 1, light exposure led to the gradual activation of HO-1 from 1 to 3 days. The level of HO-1 was significantly elevated, even on the first day of light exposure, compared with the level in the dark control group (P<0.05), and reached a peak on the third day. Reduced glutathione (GSH) and oxidized glutathione (GSSG) make up an important intracellular defense system for anti-oxidation, thus GSH and GSSG levels were determined, and the ratio of GSH/GSSG was calculated. As shown in Figure 2B, the ratio of GSH/GSSG was significantly decreased on the third day after light exposure compared with the GSH/GSSG ratio in the dark control group (P<0.05), suggesting a severe imbalanced redox status in the cells caused by light exposure. In addition, to further verify the role of oxidative stress in the death pathway, the protective effect of suppressing oxidative stress on light-damaged cells was examined using the antioxidant, NAC. As shown in Figure 2A and 2C, NAC treatment (5 mM for 661W cells and 2.5 mM for ARPE-19 cells) significantly reduced intracellular ROS generation and the level of HO-1, but increased the ratio of GSH/GSSG on the third day of light exposure compared to the vehicle group (P<0.05). Most importantly, treatment with NAC (5 mM for 661W cells and 2.5 mM for ARPE-19 cells) significantly attenuated the percentage of cell death caused by light damage compared with the light-damaged vehicle group (P<0.05; Figure 2D). Taken together, these results suggest that light exposure leads to severe oxidative-stress injury in photoreceptors and RPE cells, and may function as an upstream step triggering the subsequent activation of the death cascade.

### Light exposure induces ER stress in photoreceptors and RPE cells

**Figure 3:**
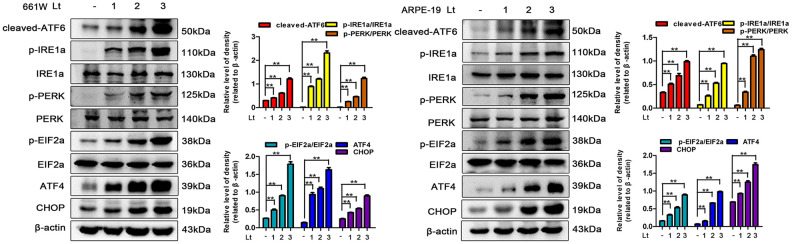
**Light exposure induces ER stress in photoreceptors and RPEs.** 661W cells and ARPE-19 cells were cultured in a dark condition or exposed to 1500 Lux light for 1–3 days after which the levels of ER stress markers were determined by western blotting. β-actin was referenced as an internal control. Three independent experiments are conducted two weeks apart. The results are presented as the mean± SEM. n (per group) =3, **P < 0.01.

**Figure 4:**
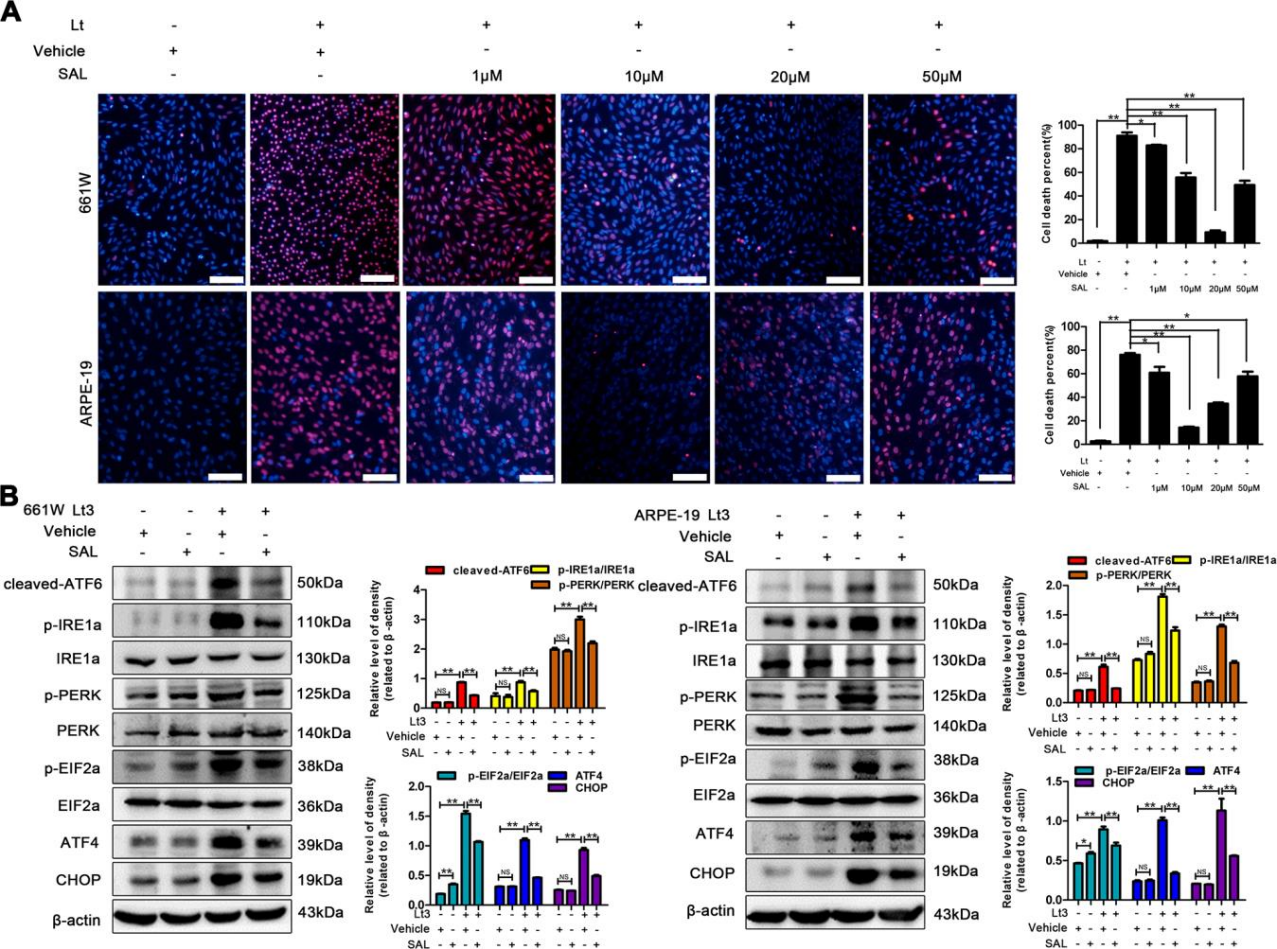
**SAL treatment suppresses light-induced ER stress and protects photoreceptors and RPEs.** (**A**) 661W cells/RPE cells were treated with SAL (1 μM, 10 μM, 20 μM and 50 μM) and cultured under 1500 Lux light or dark conditions for the indicated times. The percentage of cell death was evaluated with PI/Hoechst staining. Scale bar=100 μm. (**B**) The cells were treated with SAL (20 μM for 661W cells; 10 μM for ARPE-19 cells) or vehicle and cultured under light/dark conditions for 3 days, after which the levels of ER stress markers in the whole cell lysate were determined with western blotting, and β-actin was referenced as an internal control. Three independent experiments are conducted two weeks apart. The results are presented as the mean± SEM. n (per group) =3, NS: no significance, *P < 0.05, **P < 0.01.

**Figure 5:**
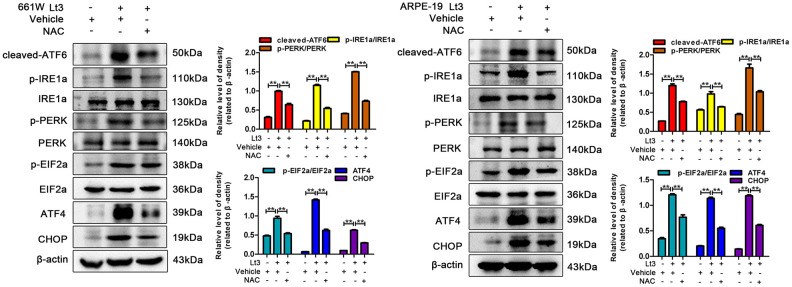
**NAC treatment suppresses light-induced ER stress in photoreceptors and RPEs.** The cells were treated with NAC (5 mM for 661W cells; 2.5 mM for ARPE-19 cells) or vehicle and cultured under light/dark conditions for 3 days, the levels of ER stress markers were determined with western blotting, and β-actin was referenced as an internal control. Three independent experiments are conducted two weeks apart. The results are presented as the mean± SEM. n (per group) =3, **P < 0.01.

Excessive ROS in cells may lead to depletion of the GSH pool and compromise of the function of PDI, which disrupts the folding process of proteins in the ER, causing the accumulation of unfolded or misfolded proteins and triggering ER stress [16]. Therefore, the markers in three signal pathways of ER stress were determined after 661W and ARPE-19 cells were exposed to 1500 Lux light for 1–3 days. As shown in Figure 3, light exposure significantly caused activation of cleaved-ATF6, p-IRE1a/IRE1a, p-PERK/PERK, p-EIF2a/EIF2a, ATF4 and CHOP compared with the dark control group. The levels of these markers reached a peak on the third day of light exposure indicating that light exposure indeed induces ER stress in photoreceptors and RPE cells. To further assess the role of ER stress in the light-induced cell death pathway, salubrinal (SAL), an ER stress inhibitor, was used to suppress ER stress under light-exposure conditions. As shown in Figure 4A, treatments with SAL (1 μM, 10 μM, 20 μM, and 50 μM) were protective against light damage compared with the vehicle group, as determined using the PI/Hoechst staining assay. The optimum SAL concentration, which provided the best protection against light damage and led to the lowest cell death rate was around 20 μM for 661W cells and 10 μM for ARPE-19s. Treatment with SAL (20 μM for 661W cells and 10 μM for ARPE-19s) consistently suppressed the activation of ER stress, reducing the levels of cleaved-ATF6, p-IRE1a/IRE1a, p-PERK/PERK, p-EIF2a/EIF2a, ATF4 and CHOP after 3 days of light exposure compared with the light vehicle group (Figure 4B). However, treatment with SAL under dark conditions caused a slight increase in p-EIF2a (P<0.05, vs dark vehicle group), suggesting that SAL is pharma-cologically functional since SAL treatment may prevent EIF2α dephosphorylation by inhibiting the protein complex GADD34/protein phosphatase 1 (PP1) [36]. The correlation between oxidative stress and ER stress under the light-exposed condition was then examined. As shown in Figure 5, treatment with the antioxidant NAC (5 mM for 661W cells and 2.5 mM for ARPE-19s) significantly suppressed ER stress, markedly reducing the levels of cleaved-ATF6, p-IRE1a/IRE1a, p-PERK/PERK, p-EIF2a/EIF2a, ATF4 and CHOP on the third day of light exposure compared with the vehicle group, which suggests that light induced-oxidative stress might be the upstream step, prior to ER stress, in the death cascade.

### Autophagy is over-activated in cells under light conditions

**Figure 6:**
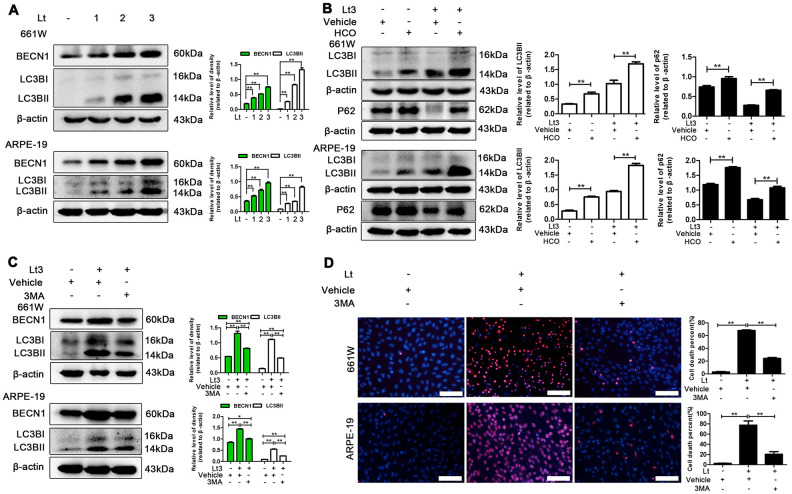
**Inhibiting light-induced prolonged autophagy is protective.** (**A**) 661W cells/ARPE-19 cells were cultured in a dark condition or exposed to 1500 Lux light for 1–3 days. The levels of BECN1 and LC3BII in the whole cell lysate was determined with western blotting, and β-actin was referenced as an internal control. (**B**) After 661W cells and ARPE-19 cells were treated with HCO (20 μM) or vehicle and cultured under light/dark conditions for 3 days, the level of LC3BII and P62 in the whole cell lysate were determined with western blotting, and β-actin was referenced as an internal control. (**C**) The cells were treated with 3MA (2.5 mM for 661W cells; 1 mM for ARPE-19) or vehicle and cultured under light/dark conditions for 3 days. The level of BECN1 and LC3BII in the whole cell lysate were determined with western blotting, and β-actin was referenced as an internal control. (**D**) 661W cells pretreated with 2.5 mM 3MA/vehicle were cultured under light/dark conditions for 3 days. ARPE-19 cells pretreated with 1 mM 3MA/vehicle were cultured under light/dark conditions for 6 days. The percentage of cell death was evaluated with PI/Hoechst staining. Scale bar=100 μm. Three independent experiments are conducted two weeks apart. The results are presented as the mean± SEM. n (per group) =3, *P < 0.05, **P < 0.01.

Prolonged ER stress may trigger autophagy-lysosome-dependent ERAD to remove accumulated abnormal proteins and protein aggregates thus, light-induced autophagy in photoreceptors and RPEs was further investigated. BECN1 is a widely used marker for assessing autophagy because it participates in the initial stage of autophagosome formation [37]. The transformation from LC3BI to LC3BII is another important process employed in the study of autophagy activation [38]. Klionsky et al suggested LC3BII /β-actin as an indicator for detecting autophagy [39]. As shown in Figure 6A, after 661W and ARPE-19 cells were exposed to light for 1–3 days, the levels of BECN1 and LC3BII in the light-damaged group significantly increased in a dose-dependent manner compared with the dark control group (P<0.05). In addition, the autophagic flux caused by light exposure was monitored. Hydroxychloroquine (HCO) may compromise the acidity of lysosomes. This interrupts autophagic clearance, which may cause the accumulation of LC3BII if the autophagic flux is blocked [38]. As shown in Figure 6B, treatment with 20 μM HCO remarkably caused the accumulation of LC3BII in 661W cells and ARPE-19s under the light-exposure condition compared with the light vehicle group (P<0.05), indicating that light exposure induces a complete autophagic process, which may be blocked by HCO treatment. In addition, p62, another marker protein of autophagy, whose expression level was negatively correlated with autophagy flux, was detected [40–42]. As shown in Figure 6B, the level of p62 in the light damaged group was significantly lower than in the dark control group, and HCO treatment was able to attenuate the decrease of p62 under the light exposure condition, indicating that light exposure induced autophagy and increased autophagy flux. Next, the role of autophagy in the light-induced death cascade was investigated. As shown in Figure 6C, treatment with 3-methyladenine (3MA; 2.5 mM for 661W cells, 1 mM for ARPE-19 cells) significantly reduced the levels of BECN1 and LC3BII in the cells exposed to light for 3 days compared with the light vehicle group and remarkably reduced the cell death rate assessed by PI/Hoechst staining under the light-exposed condition, as shown in Figure 6D. However, the levels of BECN1 and LC3BII in the 3MA-treated light-exposed group were still slightly higher than those in the vehicle dark group, suggesting that 3MA treatment simply suppresses over-activated autophagy caused by light exposure.

### Inhibiting ER stress suppresses autophagy and protects cells under light conditions

**Figure 7:**
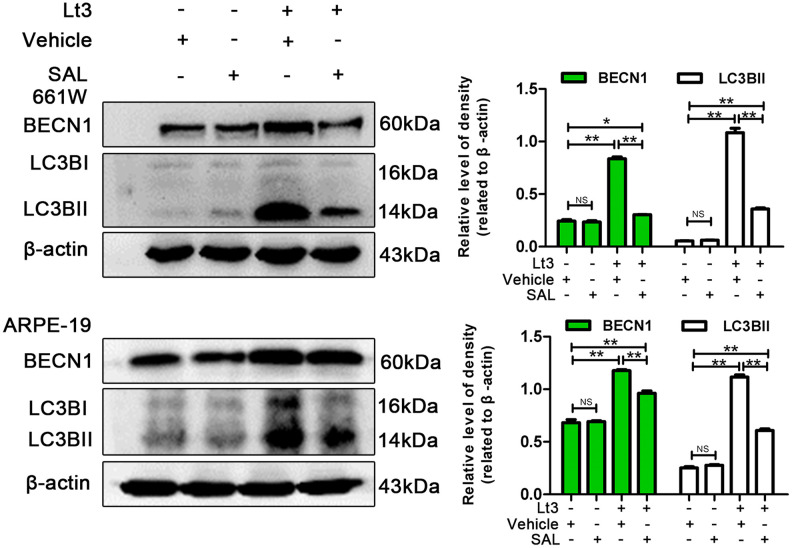
**Inhibiting ER stress suppresses light-related autophagy.** 661W cells/ARPE-19 cells were treated with SAL (20 μM for 661W cells; 10 μM for ARPE-19 cells) or vehicle and cultured under light/dark conditions for 3 days. The level of BECN1 and LC3BII in the whole cell lysate were determined with western blotting, and β-actin was referenced as an internal control. Three independent experiments are conducted two weeks apart. The results are presented as the mean± SEM. n (per group) =3, NS: no significance, *P < 0.05, **P < 0.01.

**Figure 8:**
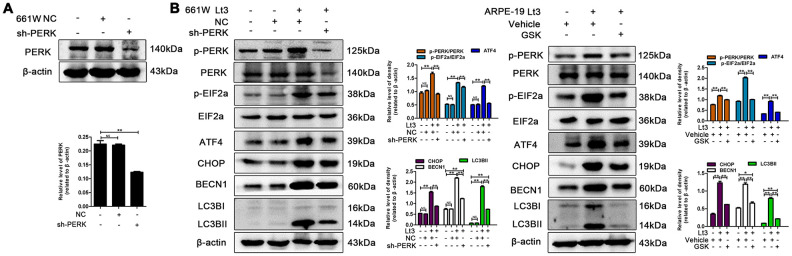
**Inhibiting PERK suppresses light-related autophagy.** (**A**) 661W cells were infected with lentivirus-expressed PERK shRNA (sh-PERK) or negative control shRNA (NC). The level of PERK in the whole cell lysate was determined with western blotting, and β-actin was referenced as an internal control. (**B**) 661W cells with stable PERK knockdown/ARPE-19 cells treated with GSK (5 μM) or vehicle were cultured under light/dark conditions for 3 days. The target proteins in the whole cell lysate were determined with western blotting, and β-actin was referenced as an internal control. Three independent experiments are conducted two weeks apart. The results are presented as the mean± SEM. n (per group) =3, NS: no significance, *P < 0.05, **P < 0.01.

**Figure 9:**
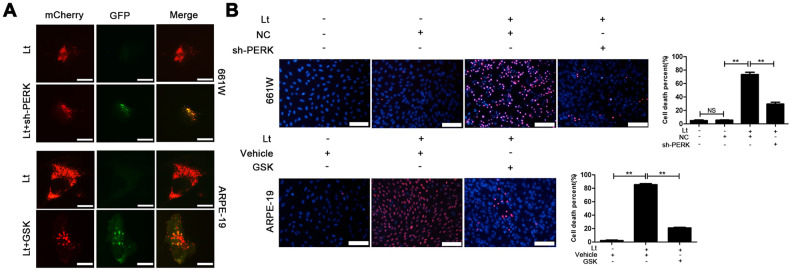
**Inhibiting PERK blocks autophagic flow and protects the light-damaged cells.** (**A**) 661W cells with stable PERK knockdown and ARPE-19 cells were infected with mCherry-GFP double labeled-LC3B mediated by adenovirus. At 48 h after infection, the ARPE-19 cells were treated with GSK (5 μM) or vehicle. The cells were cultured under 1500 Lux light condition for 3 days and photographed under fluorescence microscopy. Scale bar=20 μm. (**B**) 661W cells with PERK knockdown were cultured under light/dark conditions for 3 days, but ARPE-19 cells treated with GSK (5 μM) or vehicle were cultured under light/dark conditions for 6 days. The percentage of cell death was evaluated with PI/Hoechst staining. Scale bar=100 μm. Three independent experiments are conducted two weeks apart. The results are presented as the mean± SEM. n (per group) =3, NS: no significance, **P < 0.01.

To further verify whether light exposure induces ER stress-related autophagy, ER stress was further suppressed with SAL and the influence of ER stress on autophagy activation was examined. As shown in Figure 7, treatment with SAL significantly reduced the levels of BECN1 and LC3BII compared with the vehicle light damaged group (P<0.05), indicating that suppressing ER stress may block the light-induced activation of autophagy. Previous studies have shown that the PERK signal may participate in activation of ER-related autophagy. Therefore, in the current study, PERK activity was suppressed using one of the following two methods: knocking down PERK expression with lentivirus-mediated shRNA in 661W cells or treatment with GSK2606414 (GSK), a specific inhibitor of PERK in ARPE-19 cells. As shown in Figure 8A, the expression of PERK in 661W cells was significantly knocked down by the specific shRNA (sh-PERK) compared with the negative control (NC) group (P<0.05). In addition, activation of the downstream factors of the PERK pathway (p-PERK/PERK, p-EIF2a/EIF2a, ATF4 and CHOP) was also markedly suppressed under the light-exposed condition. Similarly, treatment with GSK also significantly suppressed activation of the PERK signal in RPE cells under the light-exposed condition, as shown in Figure 8B, causing obvious reductions in the levels of p-PERK/PERK, p-EIF2a/EIF2a, ATF4 and CHOP compared with the vehicle light damaged group. Next, the influence of PERK inhibition on the activation of autophagy was assessed. Figure 8B shows that both PERK knockdown and GSK treatment caused significant reduction in the levels of BECN1 and LC3BII in light-exposed cells compared with the light vehicle group (P<0.05). Furthermore, the influence of PERK inhibition on light-induced autophagy flux was evaluated by tracking mCherry-GFP-LC3B double labeled autophagosomes expressed by adenoviruses. The green fluorescence of GFP-labeled LC3B is quenched due to the acidic environment after autophagosomes integrate with lysosomes. Therefore, the autolysosomes with mCherry-GFP double labeled-LC3B simply turn red (the color of cherries). However, when autophagy is blocked, the number of autolysosomes is reduced and some autophagosomes with mCherry-GFP double labeled-LC3B show the overlaid yellow color. As shown in Figure 9A, the number of autolysosomes was greatly reduced in the cells with PERK inhibition. Some autophagosomes show the overlaid yellow color, yet a large number of autolysosomes in the control cells show the red color, indicating that either PERK knockdown or GSK treatment may block autophagy. Importantly, PERK knockdown and GSK treatment significantly reduced the death rate in 661W and ARPE-19 cells in the light-exposed condition as evaluated with PI/Hoechst staining, as shown in Figure 9B. Taken together, these results suggest that inhibiting ER stress via PERK signal may suppress light-induced prolonged autophagy, and that inhibiting ER stress-related autophagy protects photoreceptors and RPE cells against light damage.

### Suppressing ER stress inhibits prolonged autophagy and protects the retina against light injury

**Figure 10:**
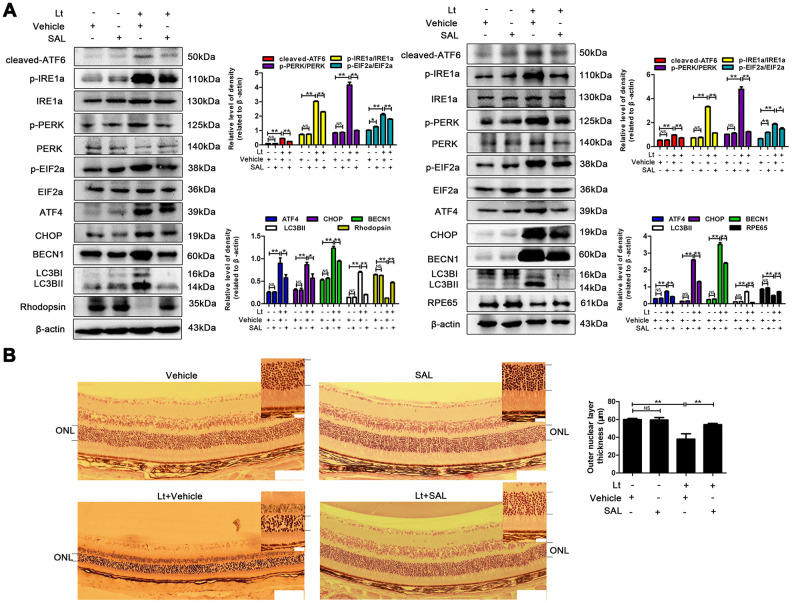
**Suppressing ER stress inhibits prolonged autophagy and protects the retina against light injury.** The mice were intraperitoneally injected with a dose of 1 mg/kg once a day for 7 days. On the third day of administration, the mice were exposed to continuous 7000 Lux visible light for 12 h. After light exposure, the mice were fed in the animal room with the normal light/dark cycle. On the fifth day of light exposure, the mice were sacrificed, and the eyeballs were enucleated. (**A**) The retinas were collected, and target proteins were determined with western blotting. β-actin was referenced as an internal control. Three independent experiments are conducted three weeks apart. The results are presented as the mean± SEM. n (per group) =3, NS: no significance, *P < 0.05, **P < 0.01. (**B**) The retinas were sectioned and stained with H&E and photographed under a microscope. Scale bar=100 μm; 20 μm. The thickness of the outer nuclear layer (ONL) was measured and quantitatively analyzed. The results are presented as the mean± SEM, n (per group) =6, NS: no significance, **P < 0.01.

Next, ER stress-related autophagy was verified in light-injured retinas of mice. As shown in Figure 10A, intensive light exposure for 12 h caused a significant increase in the levels of ER stress markers, including cleaved-ATF6, p-IRE1a/IRE1a, p-PERK/PERK, p-EIF2a/EIF2a, ATF4 and CHOP, and elevated the levels of autophagy markers BECN1 and LC3BII in both the retina and RPE/choroid samples compared to the control samples as determined with western blot. However, intraperitoneal injection of SAL significantly suppressed the light-induced activation of ER stress and autophagy, resulting in reduced levels of cleaved-ATF6, p-IRE1a/IRE1a, p-PERK/PERK, p-EIF2a/EIF2a, ATF4, CHOP, BECN1 and LC3BII compared with the light-damaged vehicle group (P<0.05; Figure 10A). In addition, light-induced retinal injury was quantitatively evaluated by measuring the level of rhodopsin, a marker of photoreceptors, and RPE65, a marker of RPE cells. Figure 10A shows that intensive light exposure resulted in marked damage to photoreceptors and RPEs, significantly reducing the levels of rhodopsin and RPE65, while SAL injection markedly attenuated the decreased levels of these two markers compared with the light-damaged vehicle group ((P<0.05). Moreover, histological analysis showed that light exposure caused obvious structural disorders in the ONL of the retina and significantly reduced the thickness of the ONL, while SAL injection attenuated the light-induced decrease of ONL thickness and maintained the normal structure of the retina (Figure 10B). These results suggest that excessive light exposure may cause ER stress and prolonged autophagy in the retina; however, suppressing ER stress may inhibit over-activated autophagy and protect the retina against light injury.

## DISCUSSION

The death of photoreceptors/RPEs is the principal event in the pathogenesis of AMD, and it is known that the excessive exposure to natural or unnatural light may accelerate this death or the death process [8]. Schick reported that sunlight exposure during working life is an important risk factor for AMD [43] and Sui reported that individuals with more sunlight exposure are at a significantly increased risk of AMD [11]. Photoreceptors and RPE cells are rich in photosensitizers [3, 44]. The excessive electromagnetic energy of photons is absorbed by these photosensitizers, which may break the bonds of other molecules via direct electron exchange or hydrogen exchange and generate excessive free radicals [2, 3]. In this study, oxidative-stress damage caused by visible light exposure in photoreceptors (661W cells) and RPE cells (ARPE-19 cells) was verified by the increased HO-1 and the decreased GSH/GSSG ratio. These results suggest that light exposure led to a severe redox imbalance in the cells and that suppressing oxidative stress with the antioxidant NAC protects photoreceptors and RPE cells against light damage. Light-induced oxidative damage might be an important step in the pathogenesis of light-related retinal diseases, especially in AMD, in which increased reactive oxygen species (ROS) production, and mitochondria dysfunction are observed together with increased abnormal protein aggregation and inflammation in the photoreceptor/RPE layer, leading to oxidative stress-induced damage to the retinal pigment epithelium which is considered to be a key factor in the pathogenesis of AMD [45].

When the cells are undergoing oxidative stress, the chaperones available for protein folding are inactivated, and the disulfide bond reduction needed for ER-associated degradation is impeded. Together, these may lead to accumulation of unfolded or misfolded proteins and trigger UPR [46]. In the current study, it was demonstrated that continuous light exposure markedly induced the activation of UPR both *in vitro* and *in vivo*. The levels of key factors in the UPR signal pathway, including cleaved-ATF6, p-IRE1a/IRE1a, p-PERK/PERK, p-EIF2a/EIF2a, ATF4 and CHOP, were significantly increased in both photoreceptors/RPE and the retina after visible light exposure. In addition, treatment with the ER stress inhibitor SAL significantly suppressed the elevated levels of ER stress markers and attenuated light-induced cell death and injury in the retina. To verify the relationship between the oxidative stress and ER stress, the influence of the antioxidant, NAC on UPR signals was further investigated. The results showed that treatment with NAC may significantly suppress the light-induced activation of ER stress, indicating that photo-oxidation might be the upstream step prior to the activation of UPR in the light-induced death cascade in photoreceptors/RPEs.

Moderate UPR is beneficial for sustaining cellular homeostasis by reducing the synthesis of unfolded or misfolded proteins. However, prolonged UPR may trigger the downstream death signal, leading cells to a programmed death [18]. Previous studies have reported that autophagy is involved in the pathogenesis of light-related retina degeneration diseases, such as AMD. Therefore, the light-induced cell death pattern and the role of autophagy in the light-induced death cascade was examined and activation of autophagy was detected in both light-injured *in vitro* and *in vivo* models. The levels of typically autophagic markers, BECN1 and LC3BII were significantly increased after light exposure. Furthermore, the results of the LC3B turnover assay and p62 degradation assay suggest that the light-induced autophagic flux, which encompasses autophagosome formation and the late phase of autolysosome formation, in photoreceptors/RPEs increased. More importantly, it was also verified that blocking autophagy with specific inhibitor 3MA was neuroprotective against light damage in photoreceptors/RPEs. This evidence strongly suggests that autophagy is over-activated in light-damaged cells. Previous studies have shown that AMD is a multifactorial disease of the retina featured by degeneration and loss of photoreceptors and RPE cells and that autophagy is involved in its pathology. Although some studies suggest that the decline of autophagy with age contributes to the age-related disease [39], there is currently no consensus on whether autophagic activity increases or decreases with age and AMD. Kozhevnikova et al. reported that the basal level of autophagy is elevated during the early stage of retinopathy and declines at progressive stages [47]. The autophagy process is increased in two mouse models of AMD and in early-onset human AMD samples but declines in late AMD [48]. Results of the current study suggest that prolonged autophagy damages photoreceptors and RPEs in light exposure conditions, and that suppressing overactivated autophagy is neuroprotective against light injury; this may be a potential therapeutic strategy for early AMD.

The excessive accumulation of misfolded proteins and protein aggregates in cells may trigger ER stress, yet autophagy is an important mechanism for degrading abnormal proteins. Recent studies have shown that both ER stress and autophagy are involved in the pathogenesis of retinal degenerative diseases [49]. Therefore, the relationship between ER stress and autophagy in light-damaged photoreceptors/RPEs was further investigated. The results showed that either pharmacologically inhibiting PERK with GSK treatment (a specific inhibitor) or genetically knocking down PERK with lentivirus expressed shRNA significantly suppressed light-induced autophagy, reducing the level of BECN1 and LC3BII, indicating that light-induced ER stress may be closely related to autophagy and that the PERK signal may be a major pathway activating ER stress-related autophagy. ER stress-related autophagy was further verified with *in vivo* experiments. Intraperitoneal injection of SAL (an ER stress inhibitor) markedly suppressed the activation of light-induced autophagy and attenuated light-induced ONL thickening in mice retinas. Wafa et al. reported that ATF4, the downstream factor of PERK, may function as a transcription factor directly or in cooperation with CHOP increasing the expression of various autophagy-related genes, including LC3B, ATG5, Atg7, and Beclin1, in mouse embryonic fibroblasts under amino acid-starved conditions [50]. Thus, further experiments are needed to verify the more detailed signal links involved in ER stress-related autophagy in the light-induced death cascade.

In conclusion, the current study demonstrated that ER stress and autophagy are both involved in light-induced death of photoreceptors and RPE cells. As an upstream step, photo-oxidation may cause an imbalance in the cellular redox status and interrupt the folding process of proteins, further triggering ER stress in photoreceptors and RPEs. Suppressing ER stress via PERK signals may inhibit prolonged autophagy and protect photoreceptors/RPEs against light damage. Inhibiting ER stress-related autophagy is neuroprotective for retinal against light injury, which may be a potential treatment strategy for AMD.

## MATERIALS AND METHODS

### Ethics approval

All animal experiments were performed according to the Association of Research in Vision and Ophthalmology (ARVO) statement regarding the use of animals in ophthalmology and vision research. The animal experiments were performed according to the protocols approved by the Institutional Animal Care and Use Committee and the Ethics Committee of the Second Hospital of Jilin University, Changchun, China (approval number: 2018038).

### Reagents and materials

Cell culture media and additives were obtained from the HyClone Company (Beijing, China). N-acetyl-L-cysteine (>99%), Salubrinal, Hoechst/PI and a Reactive Oxygen Species Assay Kit were purchased from Beyotime Biotechnology (Shanghai, China). The 3-methyladenine was purchased from Sigma-Aldrich (Shanghai, China). Hydroxychloroquine was purchased from Tokyo Chemical Industry (Shanghai, China). GSK2606414 (>99.38) was purchased from Med Chen Express (New Zealand, USA). PERK (Cat. No. 3192), IRE1a (Cat. No. 3294) and p62(Cat. No. 5114) antibodies were purchased from Cell Signaling Technology (Shanghai, China). EIF2a (Cat. No.sc-133132) antibodies were acquired from Santa Cruz Biotechnology (Beijing, China). Rhodopsin (Cat. No. OM186133) antibodies were purchased from OmnimAbs (Shanghai, China). LC3B (Cat. No. ab192890) antibodies were purchased from Abcam (Cambridge, MA, USA). BECN1 (Cat. No. 11306-1-AP) antibodies were purchased from Proteintech (Wuhan, China). P-IRE1a (Cat. No. 13013), p-PERK (Cat. No. 12814), ATF6 (Cat. No. 24382), p-EIF2a (Cat. No. 11279), ATF4 (Cat. No. 32007), CHOP (Cat. No. 40744), RPE65(Cat. No. 49495), β-actin (Cat. No. 21800) and secondary antibodies were obtained from Signalway Technology (St. Louis, MO, USA).

### Cell culture

The 661W cell line was a gift from Dr. Muayyad Al-Ubaidi (University of Oklahoma Health Sciences Center, USA). The 661W cells were grown in Dulbecco’s modified Eagle’s medium (DMEM) supplemented with 10% heat-inactivated fetal bovine serum, 100 U/ml penicillin and 100 mg/ml streptomycin. ARPE-19 cells were purchased from the American Type Culture Collection (ATCC, USA) and maintained in Dulbecco’s modified Eagle’s medium/nutrient mixture F-12 (DMEM/F-12) supplemented with 10% FBS, 100 U/ml penicillin and 100 mg/ml streptomycin. The 661W cells were passaged by 0.05% trypsin–EDTA every 2–3 days. ARPE-19 was passaged by trypsin with 0.05% EDTA every 3–4 days. The cells were cultured in a humidified atmosphere of 95% air and 5% CO^2^ at 37°C. Cell culture medium and additives were purchased from the HyClone Company (Beijing, China)

### Visual light exposure

A standard 8-W fluorescent strip light was fixed in the incubator and covered with a filter to ensure that the cells were exposed to visible light (400–800 nm), and the distance between the light source and plates was 20 cm to ensure that all cells received the same intensity of light (1500 Lux, measured with a digital light meter, TES-1332A, Taipei, China). The light exposure experiment was performed as previously described [33]. Briefly, the cells were precultured in 96 or 6 well plates for 24 h, and light exposure was started until the cell confluence reached 75%. For the dark control group, a paper box was placed in the same incubator to create a dark chamber. The cell culture medium was replaced every two days, and the temperature of the culture medium under dark or light conditions was maintained; no substantial difference in temperature was found between the two groups.

### PERK knockdown with lentiviral-mediated short hairpin RNA (shRNA)

The lentivirus expressed short hairpin RNA (shRNA) targeting the PERK gene was constructed by GeneCopoeia (Shanghai, China), and the lentivirus-mediated scrambled shRNA was produced as a negative control. The interfering sequence specifically targeting the PERK gene was as follows: forward sequence 5′-CAGGTCCTTGGTAATCATTTCAAGAGAATGATTACCAAGGACCTG-3′. The lentivirus particle was produced according to a previous protocol [51]. Briefly, a third-generation lentiviral package system was used to package lentivirus particles. Three auxiliary plasmids (pRRE, pRSV-Rev and pCMV-VSVG) and the core plasmid were mixed with lipo6000 transfection reagent (Beyotime Biotechnology, Shanghai, China) in proportion and then transfected into human embryonic kidney 293T (HEK293T) cells. After 72 h, virus particles were collected, and the virus particles were concentrated. The 661W cells were infected with the concentrated virus particles. To avoid toxicity, the virus medium was replaced with fresh complete DMEM medium 24 h after infection. To obtain cells with stable PERK knockdown, the cells were treated with puromycin (Beyotime, Biotechnology, Shanghai, China) at an initial concentration of 8 μg/mL three days after the infection, and the puromycin containing medium was replaced with fresh complete medium 48 h after the screening program. After repeating the screening program three times, cell clones with stable PERK knockdown were obtained.

### Autophagic flux measurement

The cells were infected with adenovirus-mediated mCherry-GFP-LC3B (Beyotime Biotechnology, Shanghai, China) to monitor autophagic flux, according to the commercial instructions. Briefly, the cells were precultured in a 96-well plate for 24 h, and then the medium was replaced with virus-fresh medium mixture (2 μl/100 μl). After culturing for 24 h, the virus medium was replaced with complete fresh medium, and the cells were further cultured for 24 h. After infection, the cells were exposed to the light condition, and then they were observed and photographed under an inverted fluorescence microscope (Olympus, Japan).

### Propidium iodide (PI)/Hoechst staining

The cells were stained with Hoechst 33258 dye (2 μg/mL, Beyotime Biotechnology, Shanghai, China) for 30 min in the dark at 37°C, after which the cells were stained with PI (Beyotime Biotechnology, Shanghai, China) at a final concentration of 5 μg/mL for 10 min in the dark at 4°C. The images were photographed under an inverted microscope (Olympus, Japan), and the images were quantitatively analyzed with Image J software (v1.51, NIH, USA). The cell death rate = PI-positive cells/total cells%.

### Intracellular ROS measurement

The intracellular ROS level was determined with a dichloro-dihydro-fluorescein diacetate (DCFH-DA, Beyotime Biotechnology, Shanghai, China) staining assay. Briefly, the cells were precultured in 96-well plates for 24 h. Next, the cells were washed twice with fresh medium and then cultured with FBS-free medium containing 10 μM DCFH-DA for 20 min at 37°C in the dark. After washing twice with serum-free medium, the cells were observed and photographed under a fluorescence microscope (Olympus, Japan). Fluorescence intensities were quantitatively determined with ImageJ software (v1.51, NIH, USA). All of the images were converted to grayscale images and then inverted and calibrated while using ImageJ, and the mean fluorescence intensity was obtained by dividing the gray value of all pixels in the selected area by the number of pixels [52, 53]. The relative intensity of the fluorescence was calculated as a percentage of the fluorescence intensity of the vehicle cells.

### Measurement of GSH/GSSG

The intracellular reduced/oxidized glutathione was quantitatively determined according to the commercial instructions of the GSH/GSSG Kit (Beyotime, Shanghai, China). Briefly, after 3 days of light exposure, the cells were washed twice with PBS and then harvested with a scraper and centrifuged at 1,000 rpm for 5 min at 4°C. The cell pellet was further resolved with reagent A (removing the cellular proteins) and frozen-thawed twice in liquid nitrogen and a 37°C water bath. The supernatant was collected after the cell mixture was centrifuged at 10,000 g for 10 min at 4°C for determining total glutathione and oxidized glutathione. To measure the total glutathione, solution B (containing GSH reductase, 5,5′-dithiobis-2-nitrobenzoic acid) was mixed with the supernatant and incubated at 25°C for 5 min. Then NADPH was added to the mixture to obtain a color reaction. Similarly, to measure GSSG, the cellular GSH was removed with a GSH scavenging reagent, and then the GSSG was determined following the above procedure. The absorbance at 412 nm was measured with a microplate reader (Tecan, Mannedorf, Switzerland). The concentrations of total glutathione and GSSG were calculated from the standard curve. The ratio of GSH/GSSG was calculated with the formula, GSH/GSSG = (Total glutathione-GSSG×2)/GSSG%

### Western blot analysis

Cell, retina and RPE/choroid mixture samples were sonicated in protein lysate buffer (Beyotime, Shanghai, China) containing 1% protease inhibitor cocktails (Beyotime, Shanghai, China). A bicinchoninic acid assay was used to measure the protein concentration. An equal amount (20 μg) of cell lysate was dissolved in the sample buffer, after which samples were boiled for 6 min. After denaturation, electrophoresis was performed with 10% polyacrylamide gels containing 0.1% SDS, and then proteins were transferred to nitrocellulose membranes. The membranes were blocked with 5% non-fat dry milk in Tris-buffered saline with 0.1% Tween-20 (TBS-T) for 1 h at room temperature and rinsed in TBS-T three times. Then the membranes were subsequently incubated with the specific primary antibody overnight at 4°C. The membranes were washed three times with TBS-T and incubated with the corresponding biotinylated secondary antibodies for 1 h at room temperature. Signals were subsequently developed using enhanced chemiluminescence, after which images were captured using a microscope equipped with a CCD camera (Tanon, Shanghai). Finally, the band density of proteins was calculated with the ImageJ software (v1.51, NIH, USA).

### Animals

Six-week-old male C57BL/6J mice were purchased from the Animal Center of Jilin University (Changchun) and maintained with free intake of food and water, the indoor temperature was maintained at 21°C–23°C, and a 12 h light/dark cycle was guaranteed.

### SAL treatment and light exposure protocol

The mice were divided into four groups: vehicle group (n=6), SAL-treated group (n=6), light-damaged vehicle group (n=6), and light-damaged SAL-treated group (n=6). SAL (dissolved in 1% DMSO saline) was intraperitoneally injected with a dose of 1 mg/kg once a day for 7 days. On the third day of administration, the pupils were dilated with 1% Atropine eye drops, and the mice were exposed to continuous 7000 Lux visible light for 12 h. After light exposure, the mice were fed in the animal room with the normal light/dark cycle. On the fifth day of light exposure, the mice were sacrificed by intraperitoneal injection of excessive pentobarbital sodium, and the eyeballs were enucleated. One eye was sectioned for further histological analysis, and another eye’s retina was collected for western blot analysis.

### RPE/choroid mixture isolation

The RPE/choroid mixture was separated according to a previous protocol [54]. Briefly, the cornea and lens from enucleated eyeballs were carefully removed under a surgery microscope, and then the retina and the soft tissue surrounding the sclera were removed. After sonication in RIPA lysis buffer containing 1% protease inhibitor cocktails at 4°C, the sclera tissue was separated from the RPE/choroid by centrifuge at 10,000 rpm. The supernatant containing RPE/choroid proteins was collected and stored at -20°C for western blot analysis.

### Histological analysis

The enucleated eyeballs were marked at 12 o’clock with a surgical suture and fixed with 4% paraformaldehyde solution for 24 h at room temperature. After fixation, the eyeball was rinsed twice with PBS and dehydrated with gradient concentrations of alcohol. Next, the eyeballs were plastically embedded and sectioned, and the retina was sliced along the sagittal plane. The retina slice was stained with hematoxylin and eosin (H&E), and the thickness of the outer nuclear layer at 0.5 mm apart from the optic nerve head was measured with Image J software (v1.51, NIH, USA). The morphological structure of the retina and RPE was observed and photographed under a microscope.

### Statistical analysis

Statistical analysis was performed with SPSS v 23.0 (SPSS, Chicago, Illinois, USA), and each experiment was repeated at least three times. Data are expressed as the mean ± mean standard error (SEM). Levene's test was conducted to evaluate the variance between different groups for comparative statistical analysis. Based on the homogeneity of variance, the difference between the two means was evaluated by one-way ANOVA followed by Tukey test or Dunnett's T3 test. P <0.05 was considered statistically significant.
